# Mr. Morison, on the Dublin Pharmacopœ

**Published:** 1807-12

**Authors:** Thomas Morison

**Affiliations:** Surgeon, Eustace Street, Dublin


### *To the Editors of the Medical and Physical Journal.*

GENTLEMEN,

WHEN a work on any branch of science is published in these days, it is immediately seized upon by a society of anonymous critics, who have, for some years, constituted themselves a court of literary judicature. If the conduct of those learned gentlemen was tempered with candour and good nature, their labours would be highly praise-worthy, and would much assist the investigations of every studious man; but, I am sorry to say, that it too often happens that they are much more inclined to censure than approve, to cavil than discuss.

But let me here premise that this observation is meant in its utmost latitude, and not in the remotest degree levelled against any individual of those gentlemen, whose publication I am about to allude to, and for whom I entertain the purest sentiments of friendship and esteem, well grounded upon an accurate knowledge of their general professional erudition, and of the friendly disposition which one of them particularly has manifested towards me personally on repeated occasions.

In the third number of *the Dublin Medical and Physical Essays*, some strictures have been passed upon my translation of the *Pharmacopœia of the King and Queen's College of Physicians*. In this publication I am charged with error ror and with ignorance ; " on some occasions I have added to, in others I have deviated from or perverted the meaning of the original." I am not indeed so self-sufficient as to presume that any work of mine can be exempt from faults : to err is the lot of of humanity ; and under a consciousness of this I would have acquiesced in any general charge, had not its weight, and its vital import to a work on which I have employed much labour and expence, aroused me to a more attentive consideration of those severe imputations. " I have added to, deviated from, or perverted the meaning of the original." But what is the proof that is adduced of all this ? A passage or two quoted from the *College Preface,* which it seems *non verbum verbo curavi reddere fidus interpres.*—In this single part of the original work, which being no more than a vague, loose explanation of its general design, and therefore of but little consequence, I held myself perfectly at liberty to interpret it in a free paraphrastic version ; but that, in so doing, I have perverted the *important* meaning of the original, is a charge which I must positively deny.—I am arraigned for having translated the passage, *Quædam experientiâ comprobata adjecimus ;* in these terms, *we have added some new matters, being led to do so by the persuasive power of repeated and ratified experiments.* Here, to explain *four words,* twenty, forsooth, are employed : an unpardonable *addition* truly to the subject! But will any scholar say that, in this diffuse period, the meaning of the aforesaid *four comprehensive words* is perverted ? In a similar manner have I, most fallaciously, rendered this passage, *neque verba usu sancita e loco detrudere semper audeamus ;* " such terms, however, as convey *very* precise notions, and have been established by immemorial usage, are retained and preserved with inviolable respect." Here again, how is the meaning perverted ? The only difference between the original and its translation is, that the former conveys a *negative,* and the latter a *positive* meaning. The preface literally informs us, that, the College have *not always dared to push from their places words that have been sanctioned by use ;* the translation says, that *such terms have been retained and preserved inviolate.* The expressions may vary, but the essential import of the passage is the same.

I must take notice that, whilst the reviewer here prefers his accusation against me, he at the same time injuriously imputed to me a greater share of criminality than he supposes me to have been guilty of. I have said, *such terms as convey precise notions,* and not at all *such terms as convey very*

*very precise notions.* The disingenuous interpolation of the particle *very* is indeed a gross perversion of *my* meaning, and which, at the same time, gives rather a ludicrous cast to the whole sentence.—But, upon the whole, why is any particular passage of this said preface selected for the foundation of a criminal charge? Let the *whole* of it be compared with my translation, and it will be seen that I have, in every period " added to " and dilated the expressions of the original; but I defy any Pseudo-Aristarchus to prove that I have thereby *perverted* its meaning.—And further, I cannot, from strictures such as these, avoid to entertain some suspicion that my reviewer is himself not perfectly acquainted with the rules of good translation.

The terms of art, especially in Chemistry, have of late sustained so many metamorphoses, as to render it no easy matter to understand them in their various transmutations. In the best dictionaries of the Latin language, the word *argilla* signifies no more than, as its etymology implies, a *white earth,* or *clay;* but in the language of Chemistry it signifies neither.—What is it then? In God's name let it be argil, (see my appendix) or some such term, to end all disputes; assign some specific meaning to it, and in my next edition let it be so printed.

But with regard to the *cineres clavellata* not being properly translated potass, or pearl-ash, I am not inclined to yield so complaisantly, but will maintain the propriety of the version, (see the list of the materia medica.)

Whether, in the preparation of the *water of the sub-carbonate of kali,* the throat of the tunnel is to be *choaked with a linen rag,* or whether a linen cloth is directed to be *tied upon its dependent narrow orifice,* the language of the original does by no means clearly discriminate; nor is the choice of either of these modes of obturation of any manner of consequence in the process. But I will here incidentally observe, that, a little bit of clean *flannel* cloth applied in either way, would serve much better for the percolation of the fluid, being more spongy and permeable, than any linen cloth whatever.—And I will moreover, in direct opposition to the assertion of the reviewer, maintain, that my translation of this simple operation exhibits no " example of deviation from the original."

In the process of distilling the *water of caustic ammonia,* the translation of the concluding part of the operation, when given into the printer's hands, ran thus.—*Having first shaken the compound altogether, and carefully luted the vessels at their juncture, distil into a cooled receiv-*

*er,*

*er, and with a mean degree of heat, twenty ounces of the liquor.* I am happy to reflect, that the perspicacity of my Reviewer has here led me to compare my translation of this passage with the original, and to detect its errors, which the reviser of the sheets, in their progress through the impression, could not discover, this page of the manuscript having been lost in the Printing-house, and therefore not returned to him together with the proof sheet.

The words *acetous, nitrous, resinous, &c.* I have always written *acetose, nitrose, resinose, &c.* as I think that form of orthography to approach nearer to their origin than what is generally practiced. I have adopted it not from any affectation of singularity, and am certain that it never can, in any possibility, infringe the laws of chemical nomenclature.

The ambiguity of expression which the Reviewer observes in my translation of the process for the distillation of the *sulphuric ætherial liquor,* must be imputed rather to the original than to me alone. This defect did not pass unobserved by me, but my business was to *translate,* not to *reform*; and that my translation fairly represents the original, such as it is, I do positively assert. The directions for the formation of this ætherial liquor, as they at present stand in the Pharmacopœias, are given in the following terms.

℞. Spiritus vinosi rectificati, Acidi sulphurici, utriusque *pondere* uncias triginta duas. Infundatur spiritus calefactus ad gradum 120 retortæ vitreæ calori subitaneo ferendo aptæ, et continuo rivo affundatur acidum ; commisceantur paulatum, et in excipulum refrigeratum distillent liquoris (what liquor?) *mensura* uncia viginti, calore subitaneo et satis valido.

Acido in retorta residuo si affundantur spiritus vinosi rectificati unciæ sexdecim, iterum distillatione prodibit liquor æthereus sulphuricus.

If I might propose a form for the preparation of this fluid, which possibly might be found sufficiently clear and unexceptionable, I would offer it as follows.

Indetur spiritus calefactus ad gradum 120 retortæ vitreæ calori subitaneo ferendo idoneæ, et rivo perpetuo affundatur acidum ; commisceantur lente; et, adhibito calore subito et satis valido, in excipulum refrigeratum distillent liquoris ætherei sulphurici *mensura* uncia viginti.

Acido in retorta residuo si denuo affundatur spiritus vinosus rectificatus ad uncias sexdecim, et iteretur distillatio, tio, altera quinctiam exsurget portio liquoris ætherei sulphurici.

It was my original intention to have inserted in my Appendix this as well as several other proposed amendments of the Formulæ of this Pharmacopœia; but on reflection, I relinquished it, as it might be deemed an invidious measure, and a demonstration of arrogance, which I am ever most timidly anxious to avoid; and moreover, as any undertaking of that nature would have immoderately enlarged a book, already, in my apprehension, sufficiently voluminous.

The Reviewer now, "for the first time, reads in my translation, that hogs lard will *dissolve* in water;" and perhaps it may not be the last, if words shall continue to be applied in their proper signification. His conception of the term is confined to denote the perfect commiscibility, of saline substance for instance, with water, or with their proper solvent in transparent solution; but the term may, with equal propriety, be used in a more extended sense to express the separation and disunion of the cohering parts of any mass in any fluid that may be properly employed to cause such a *dissolution.*

When I had said, in the beginning of the instructions for the *preparation of hogs lard,—melt it,* &c. I might as correctly have said, *dissolve it*; but I varied the terms, perhaps with a needless nicety, to avoid a repetition of the same word.

I have given the ounce measure of distilled water, as containing 467 grains, whereas its real quantity is 457 grains. This is the mistake of the original; and who could suspect such an error in a work, in the composition of which so much time, and in the printing of which so much expence had been employed? I entered into no calculations.

My Reviewer now closes his strictures with a discommendation of *my style.* This is strange indeed after my solemn renunciation of all pretensions to the elegancies of style. For what tropes and metaphors can be invented to decorate a style employed to describe the composition of decoctions, electuaries and ointments? If my language conveys a clear meaning, it is sufficient; and into this I will not hesitate to provoke inquiry.

The Reviewer will please to accept my thanks for having pointed out a few typographical errors, the correction of which shall be duly attended to. I fear that some others, exclusive of the published list of errata, may yet be be found in the pages of this translation, owing to the precipitancy with which the work was hurried into its publication, and to my inability, through the importunity of my professional avocations, to devote that attention to it which its importance and utility demanded.

I am, &c.

THOMAS MORISON, Surgeon.

*Eustace Street, Dublin.*

